# N-terminal domain of *Bothrops asper* Myotoxin II Enhances the Activity of Endothelin Converting Enzyme-1 and Neprilysin

**DOI:** 10.1038/srep22413

**Published:** 2016-03-02

**Authors:** A. Ian Smith, Niwanthi W. Rajapakse, Oded Kleifeld, Bruno Lomonte, Nkumbu L. Sikanyika, Alexander J. Spicer, Wayne C. Hodgson, Paul J. Conroy, David H. Small, David M. Kaye, Helena C. Parkington, James C. Whisstock, Sanjaya Kuruppu

**Affiliations:** 1 Department of Biochemistry & Molecular Biology, Biomedical Discovery Institute, Monash University, Clayton, Vic 3800, Australia; 2 Baker IDI Heart and Diabetes Institute, 75 Commercial Road, Melbourne, Victoria 3004, Australia; 3 Department of Pharmacology, Biomedical Discovery Institute, Monash University, Clayton, Vic 3800, Australia; 4 Department of Physiology, Biomedical Discovery Institute, Monash University, Clayton, Vic 3800, Australia; 5 Menzies Research Institute, University of Tasmania, Hobart, Tasmania 7000, Australia; 6 Instituto Clodomiro Picado, Facultad de Microbiología, Universidad de Costa Rica, San José, Costa Rica.

## Abstract

Neprilysin (NEP) and endothelin converting enzyme-1 (ECE-1) are two enzymes that degrade amyloid beta in the brain. Currently there are no molecules to stimulate the activity of these enzymes. Here we report, the discovery and characterisation of a peptide referred to as K49-P1-20, from the venom of *Bothrops asper* which directly enhances the *activity* of both ECE-1 and NEP. This is evidenced by a 2- and 5-fold increase in the Vmax of ECE-1 and NEP respectively. The K49-P1-20 concentration required to achieve 50% of maximal stimulation (AC_50_) of ECE-1 and NEP was 1.92 ± 0.07 and 1.33 ± 0.12 μM respectively. Using BLITZ biolayer interferometry we have shown that K49-P1-20 interacts directly with each enzyme. Intrinsic fluorescence of the enzymes change in the presence of K49-P1-20 suggesting a change in conformation. ECE-1 mediated reduction in the level of endogenous soluble amyloid beta 42 in cerebrospinal fluid is significantly higher in the presence of K49-P1-20 (31 ± 4% of initial) compared with enzyme alone (11 ± 5% of initial; N = 8, *P* = 0.005, unpaired *t*-test). K49-P1-20 could be an excellent research tool to study mechanism(s) of enzyme stimulation, and a potential novel drug lead in the fight against Alzheimer’s disease.

Metalloproteases play a central role in regulating many physiological processes and consequently abnormal activity of these enzymes contribute to a wide range of disease pathologies. These include cardiovascular^1^ and neurodegenerative disease^2^ as well as many types of cancers^1^. Inhibitors of metalloproteases are widely used in research applications with some also approved for use in the clinic. However, molecules which stimulate the activity of these enzymes are rarely encountered, and as such our understanding of the mechanism(s) behind enzyme stimulation remains poor. Stimulators of enzyme activity can provide novel insights into enzyme biology and potentially open up avenues for the design of a novel class of drugs. For instance, ECE-1 and NEP are two metalloproteases that degrade amyloid beta (Aβ), the accumulation of which is a hallmark of Alzheimer’s disease.

Therefore it is of great interest to regulate the production of, and more importantly, the degradation of Aβ by stimulating the activity of these enzymes^2^. This in turn could reverse, prevent or at least halt the progression of Alzheimer’s disease.

Previous studies using animal models of Alzheimer’s disease have shown that *increasing the expression* of ECE^3^ and NEP^4^ through DNA based techniques can have beneficial effects. However, DNA based approaches can pose challenges for clinical translation. Molecules which can *directly stimulate the activity* of ECE-1 and NEP, or increase their expression are more attractive alternatives. Several studies have reported on the presence of molecules which increase the expression of or activity of NEP^5^,^6^,^7^. However, there are no reports on molecules which stimulate the activity of ECE-1. For example, polyphenols in green tea have been reported to increase the activity of NEP in cell culture models^5^, while the neuroprotective hormone humanin has been shown to increase the expression of NEP in a mouse model of Alzheimer’s disease^6^. In addition, Kynurenic acid elevates NEP expression as well as activity in human neuroblastoma cultures and mouse cortical neurones^7^. Therefore this study aimed to identify a molecule which stimulates the activity of ECE-1. Here we report on the discovery of K49-P1-20, a 20 amino acid peptide from the venom of *B. asper* which stimulates the activity of both ECE-1 and NEP. The effect of this peptide on other closely related enzymes was also examined.

## Results

### Identification of K49-P1-20

**Figure 1:**
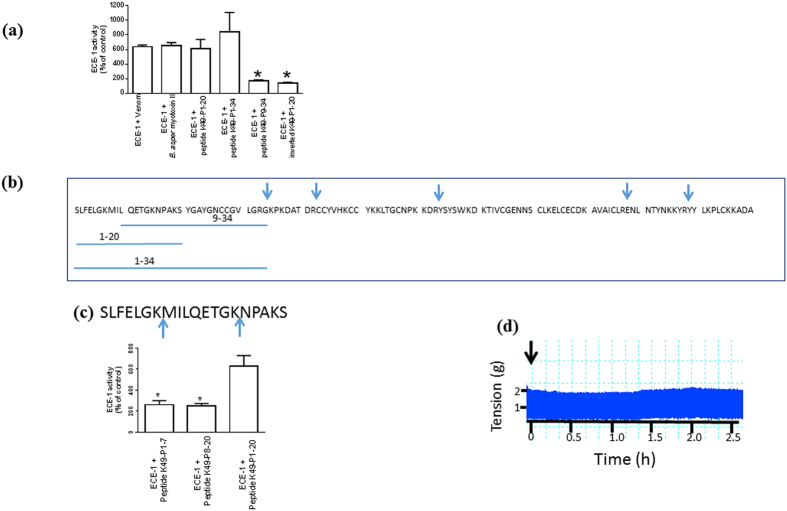
Discovery of K49-P1-20 (**a**) Comparison of ECE-1 stimulating effects of venom, *B. asper* myotoxin II, peptides K49-P1-20, K49-P1-34, K49-P9-34 and inverted K49-P1-20 (10 ng/μL); (**b**) Schematic showing the amino acid sequence of *B. asper* myotoxin II (ArgC mediated cleavage sites are indicated by arrows). The underlined sections correspond to the sequence of synthetic peptides tested for their effects on ECE-1 activity; (**c**) trypsin mediated cleavage of K49-P1-20 produces peptides K49-P1-7 and K49-P8-20 (cleavage sites indicated by arrows, top panel); the effect of K49-P1-20, peptides K49-P1-7 and K49-P8-20 on ECE-1 activity (bottom panel); (**d**) a representative trace showing the effect of K49-P1-20 (25 μg/mL) on direct twitches of the chick biventer cervices muscle. The arrow indicates the point of addition of peptide. *Significantly different than ECE-1 + peptide K49-P1-20, *P* < 0.05, unpaired *t*-test, n = 4–8.

We screened venom from species across different geographical regions for their effects on ECE-1 activity. The venom from *B. asper* was found to stimulate the activity of ECE-1 (624 ± 27% of control; Fig. 1a). Fractionation of venom confirmed that ECE-1 stimulation was mediated by the previously isolated *B. asper* myotoxin II (Fig. 1a), a lysine 49 (K49) type phospholipase A_2_ found in this venom which induces myonecrosis upon envenoming^8^. Digestion of *B. asper* myotoxin II with ArgC proteinase indicated that the stimulation of ECE-1 activity was mediated by its N-terminal region (Fig. 1a). The synthetic peptide K49-P1-34 corresponding to the N-terminal region mimicked the stimulator effects of *B. asper* myotoxin II (Fig. 1a,b). No significant difference in the activation was observed between peptides K49-P1-20 and K49-P1-34 (Fig. 1a). However, the level of stimulation observed in the presence of K49-P9-34 and inverted sequence of K49-P1-20 was significantly less compared with native K49-P1-20 (Fig. 1a). Further digestion of peptide K49-P1-20 resulted in a reduction in its ability to stimulate ECE-1 activity (Fig. 1c) indicating the importance of residues 1-20 for maximal stimulation of ECE-1 activity. Peptide K49-P1-20 failed to inhibit direct twitches of the chick biventer cervicis nerve muscle preparation, confirming its lack of myotoxic effects (Fig. 1d), in agreement with the previous mapping of toxicity determinants of *B. asper* myotoxin II to its C-terminal region^9^.

### Enzyme activity assays

**Figure 2:**
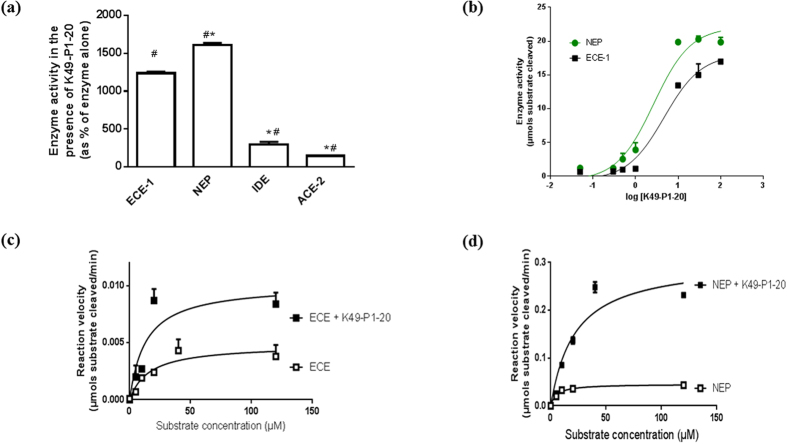
Effect of K49-P1-20 on the kinetics of ECE-1 and NEP activity (**a**) Comparison of the activity of ECE-1, NEP, IDE and ACE-2 in the presence of K49-P1-20 (10 ng/μL; *significantly different compared to ECE-1 + K49-P1-20, n = 3-4, one-way ANOVA, *P* < 0.05; and ^#^significantly different compared to enzyme alone; unpaired *t*-test, n = 3-4, *P* < 0.05); (**b**) the concentration dependant effect of K49-P1-20 on ECE-1 and NEP activity; effect of K49-P1-20 on reaction velocity of (**c**) ECE-1 and (**d**) NEP when using bradykinin based QFS as a substrate.

The presence of K49-P1-20 (10 ng/μL or 4.6 μM) significantly increased the activity of ECE-1, NEP, Insulin Degrading Enzyme (IDE) and Angiotensin Converting Enzyme-2 (ACE-2) compared to respective control. However the level of stimulation observed for IDE and ACE-2 was significantly less compared with ECE-1(Fig. 2a).

K49-P1-20 increased the activity of ECE-1 and NEP in a concentration dependant manner. Maximal stimulation of ECE-1 (1563 ± 23 as % of control) and NEP (1605 ± 58 as % of control) activity was achieved at a K49-P1-20 concentration of 100 ng/μL (or 46 μM; Fig. 2b). The activation concentration 50 (AC50) of K49-P1-20 against ECE-1 (1.92 ± 0.07 μM) is significantly higher than that against NEP (1.33 ± 0.12 μM; *P* = 0.015, n = 3; unpaired *t*-test).

### Kinetics of enzyme activation

Using a previously described assay based on a bradykinin based quenched fluorescent substrate (QFS)^10^,^11^,^12^, we examined the effect of K49-P1-20 on the kinetics of ECE and NEP. The presence of K49-P1-20 increased the Vmax of ECE-1 and NEP by 2.0 - and 5.2-fold respectively. K49-P1-20 also induced a 3.3-fold increase in Km of NEP, while significantly decreasing that of ECE-1 (Fig. 2c,d and Table 1).

### Alanine scan

**Figure 3:**
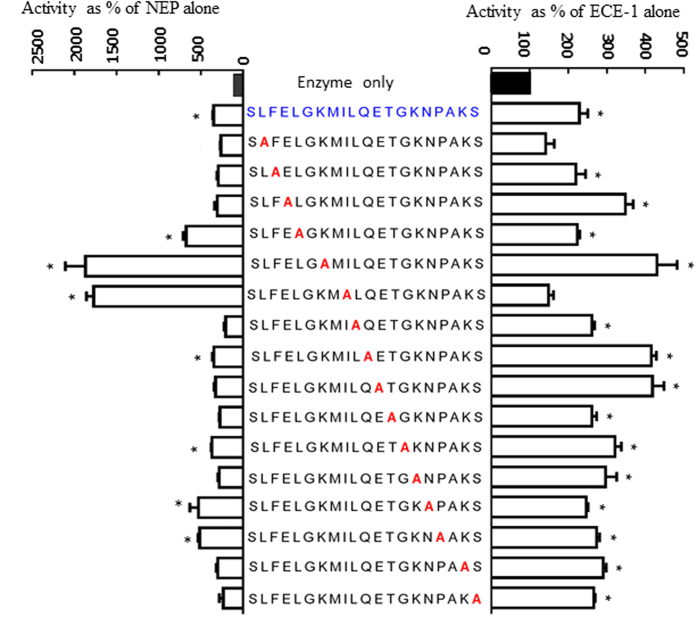
Alanine scan. A library of K49-P1-20 analogs were synthesised where each subsequent residue was replaced by an Ala. These analogs were tested for their ability to stimulate ECE-1 and NEP activity. The K49-P1-20 analogs are shown in the middle, with the Ala substitutions indicated in red. Closed bar denotes enzyme alone and the native peptide is indicated in blue *significantly different compared to enzyme alone; *P* < 0.05; One-way ANOVA; n = 4.

Alanine substitution of Leu(2) and Ile(9) failed to enhance ECE-1 activity, indicating their importance for stimulating ECE-1 (Fig. 3). Alanine substitution of Leu(2), Phe(3), Glu(4), Leu(10), Glu(12), Thr(13), Lys(15), Lys(19) and Ser(20) failed to enhance NEP activity, indicating their importance for stimulating NEP (Fig. 3).

### K49-P1-20 and enzyme interaction and conformational changes

#### BLITZ Biolayer interferometry

**Figure 4:**
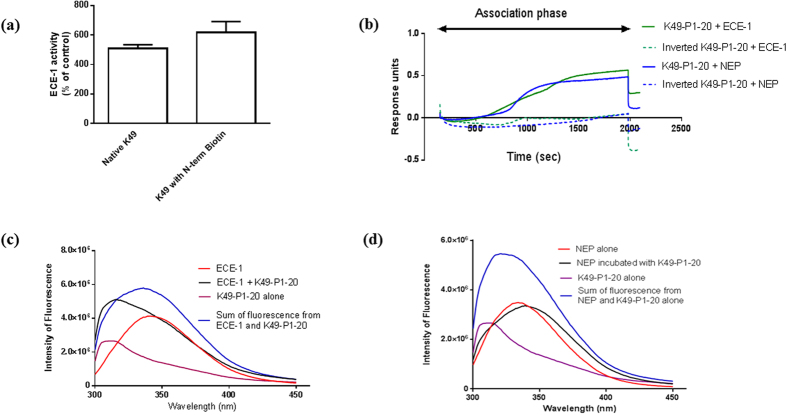
Association between K49-P1-20 and enzymes. (**a**) Effect of N-terminal biotinylation of K49-P1-20 on the activity of ECE-1. (**b**) Representative traces obtained using Biolayer interferometry showing the level of interaction between enzymes and the biotinylated version of native or inverted K49-P1-20; representative traces showing the effect of K49-P1-20 on the intrinsic fluorescence of (**c**) ECE-1 and (**d**) NEP. Fluorescence of K49-P1-20 alone, and the sum of fluorescence intensities of K49-P1-20 and enzyme is also indicated.

N-terminal biotinylation of K49-P1-20 had no significant effect on its ability to stimulate ECE-1 activity (Fig. 4a). Interaction of ECE-1 and NEP with biotinylated K49-P1-20 immobilised on a streptavidin biosensor was indicated by an increase in response units (nm) over time (Fig. 4b). The interaction was rapidly reversible. There was only a minimal interaction between each of the enzymes and biotinylated version of inverted K49-P1-20.

#### Spectroscopy

To determine whether interaction between K49-P1-20 and ECE-1 or NEP leads to a change in conformation of the native enzyme, we monitored the effect of K49-P1-20 on the intrinsic fluorescence of the two enzymes. K49-P1-20 indeed changed the intrinsic fluorescence spectra of ECE-1 and NEP, suggesting altered solvent exposure of the fluorescent amino acid residues Trp, Tyr and Phe. ECE-1 alone displayed a *λ*max of 341 nm. The presence of K49-P1-20 induced a blue shift of *λ* max to 315 nm along with a 23% increase in maximum fluorescence intensity (Fig. 4c). NEP alone displayed a *λ* max of 333 nm which was shifted by K49-P1-20 to 339 nm, with 4% reduction in the intensity of maximum fluorescence (Fig. 4d).

### Peptide cleavage assays

#### Cleavage of BigET_18–34_ and enkephalin by ECE-1 and NEP respectively

**Figure 5:**
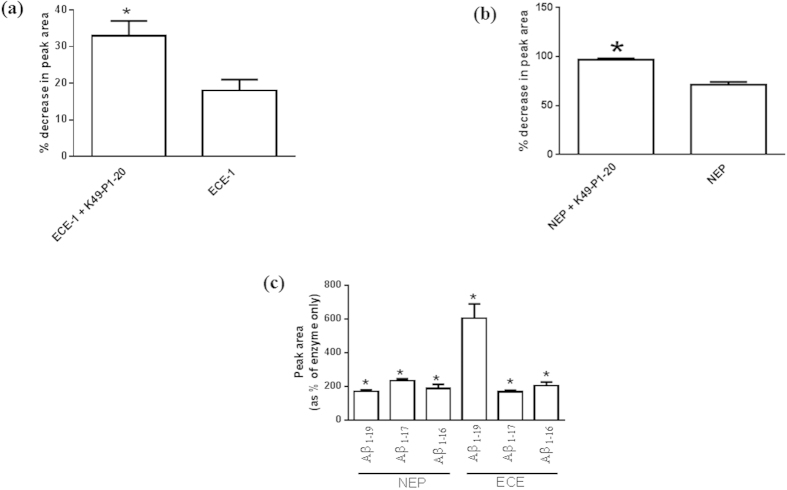
Effect of K49-P1-20 mediated enzyme activation on the cleavage of BigET_18-34_, enkephalin and synthetic Aβ40. The cleavage of substrates (**a**) BigET_18-34_ by ECE-1 and (**b**) enkephalin by NEP was monitored over 24 h using LCMS. Data are expressed as a % decrease in the amount of each substrate as indicated by peak area. (**c**) LCMS was used to monitor the formation of peptides (Aβ_1-16_, Aβ_1-17_ and Aβ_1-19_) that form as a result of ECE-1 and NEP mediated cleavage of synthetic Aβ40 over 24 h, both in the presence or absence of K49-P1-20. Data are expressed as a % of peak area corresponding to respective product obtained in the presence of enzyme only. *P < 0.05; significantly different than enzyme alone; unpaired *t*-test; n = 4.

Using LCMS we monitored the cleavage of BigET_18–34_ by ECE-1 (Fig. 5a), and enkephalin by NEP (Fig. 5b). Whilst enkephalin is a widely reported substrate for NEP^13^, BigET_18–34_ is a truncated version of the natural substrate Big Endothelin (BigET) which we have previously used to examine ECE-1 activity in cell culture media^12^ and cerebrospinal fluid^10^. ECE-1 alone induced a 18 ± 3% decrease in peak area corresponding to BigET_18–34_, while the decrease was significantly higher (33 ± 1%; unpaired *t*-test; *P* = 0.04; n = 3) in the presence of K49-P1-20.

Similarly, NEP alone induced a 71 ± 3% decrease in peak area corresponding to enkephalin. In the presence of K49-P1-20 the decrease in peak area was significantly higher at 97 ± 1% (unpaired *t*-test; *P *= 0.001; n = 3–4).

#### Cleavage of synthetic Aβ40 by ECE-1 and NEP in the presence of K49-P1-20

A potential clinical use of K49-P1-20 is the enhancement of Aβ degradation by ECE-1 and NEP so as to prevent the accumulation of harmful oligomers. We used LCMS to monitor the cleavage of synthetic Aβ40 by ECE-1 or NEP alone and in the presence of K49-P1-20. Although ECE-1 or NEP mediated cleavage of Aβ40 is known to produce several cleavage products^14^, we were only able to positively identify the products Aβ_1-16_, Aβ_1-17_, and Aβ_1-19_ (Fig. 5c). Peak area co-responding to all three products increased significantly in response to K49-P1-20. This indicates accelerated cleavage of Aβ40 by ECE-1 and NEP in the presence of K49-P1-20.

### Effect of K49-P1-20 on the activity of NEP expressed on HEK293 cells

**Figure 6:**
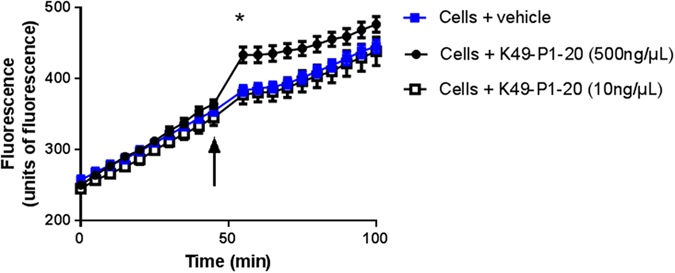
Stimulation of NEP activity in cells. Effect of vehicle or K49-P1-20 (10–500 ng/μL) on the rate of QFS cleavage by HEK293 cells. Arrow indicates the point of addition of K49-P1-20 or vehicle. *Significantly different compared to cells treated with vehicle; unpaired *t*-test, n = 4; *P* < 0.05.

The addition of K49-P1-20 (500 ng/μL) after 45 min as opposed to vehicle, significantly elevated the rate of cleavage of QFS by HEK293 cells (Fig. 6). However after 100 min, there was no significant difference in the level of fluorescence between K49-P1-20 or vehicle treated cells.

### K49-P1-20 stimulates ECE-1 activity in cerebrospinal fluid

**Figure 7:**
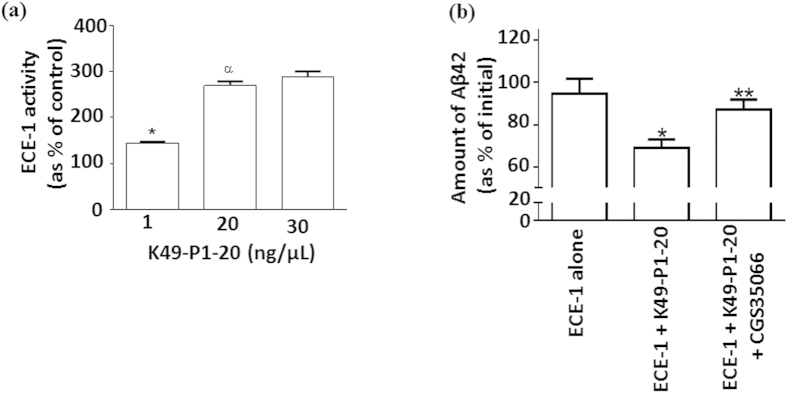
K49-P1-20 stimulates ECE-1 activity in cerebrospinal fluid (**a**) the effect of K49-P1-20 (1–30 ng/μL) on the activity of rhECE-1(0.04–ng/μL) added to cerebrospinal fluid obtained from a patient with Alzheimer’s disease at post mortem. Enzyme activity was measured using the bradykinin based QFS. * & ^α^ significantly different compared to ECE-1 alone or K49-P1-20 (1 ng/μL) respectively; *P* < 0.001; n = 5; one-way ANOVA. (**b**) The effect of ECE-1 alone (0.04 ng/μL); ECE-1 incubated with K49-P1-20 (300 ng/μL); or ECE-1+ K49-P1-20 + ECE-1 inhibitor CGS35066 (500 nM), on the levels of endogenous Aβ42 in cerebrospinal fluid taken from a patient with Alzheimer’s disease at post-mortem was determined using a commercially available ELISA kit. Significantly different compared to *ECE-1 alone *P* = 0.005; or **ECE-1 + K49-P1-20, *P* = 0.009; unpaired *t*-test, N = 8–11.

K49-P1-20 (1–30 ng/μL) stimulated the activity of rhECE-1 in cerebrospinal fluid obtained from a patient with Alzheimer’s disease, as evidenced by the enhanced cleavage of bradykinin based QFS (Fig. 7a). Addition of stimulated ECE-1 to cerebrospinal fluid obtained from patients with Alzheimer’s disease (N = 8) resulted in a significant decrease (31 ± 4%) in the levels of endogenous soluble Aβ42 over 4 h, compared with the addition of non-stimulated ECE-1 (11 ± 5%; *P* = 0.005, unpaired *t*-test Fig. 7b). This decrease was blocked by the ECE-1 specific inhibitor CGS35066 (Fig. 7b).

## Discussion

ECE-1 and NEP are two closely related metalloproteases that play a key role in many physiological and pathophysiological processes^2^,^15^,^16^. A common substrate to both enzymes is Aβ which plays a key role in the pathogenesis of Alzheimer’s disease^2^,^15^,^16^,^17^,^18^. Previous studies have reported the discovery of molecules which increase NEP activity^5^,^6^,^7^. However, there are no reports on molecules that increase ECE-1 activity. Here we report on the discovery of a peptide named K49-P1-20 from the venom of *B. asper* which stimulates the activity of both ECE-1 and NEP. Interaction of K49-P1-20 with ECE-1 or NEP appears to induce a change in its conformation leading to an increase in activity. Unlike the molecules reported in previous studies which increase NEP expression and therefore cellular NEP activity^5^,^6^,^7^, K49-P1-20 appears to allosterically regulate the activity of ECE-1 and NEP.

Animal venoms have long been a source of lead compounds for future pharmaceuticals and research tools^19^,^20^. We therefore screened venoms of snakes found in different geographical regions to identify a molecule that modulates the activity of ECE-1, and found that the venom of *B. asper* stimulated ECE-1 activity. Initial fractionation of venom indicated that this effect was mediated by a toxin known as *B. asper* myotoxin II which induces myonecrosis following envenoming^8^. *B. asper* myotoxin II belongs to a class of toxins known as Lysine 49 phospholipase A_2_ myotoxins^21^. Asp to Lys substitution at position 49 is a key structural feature of these toxins and their toxic effects are independent of the phospholipase A_2_ activity. Digestion of this toxin with ArgC proteinase indicated that stimulation of ECE-1 activity was mediated by its N-terminal domain. The use of synthetic peptides of varying length corresponding to this region confirmed that these effects were in fact mediated by its first 20 amino acids. Inverted sequence of K49-P1-20 failed to induce an increase in ECE-1 activity (136 ± 12 as % of ECE-1 alone; n = 3-4), indicating that the specific sequence of K49-P1-20 is critical for the observed effects. Further shortening of this peptide resulted in a loss of ECE-1 stimulating effects. K49-P1-20 therefore appears to possess the shortest optimum sequence required for ECE-1 stimulation and was used in all downstream studies. Previous studies have shown that myotoxic effects of *B. asper* myotoxin II are mediated by is C-terminal domain^9^. In agreement with this result, K49-P1-20 showed no myotoxicity in chick biventer cervicis muscle.

Compared with enzyme alone, K49-P1-20 also significantly enhanced the activity (expressed as % of control) of closely related enzyme NEP (1606 ± 29), and two other metalloproteases ACE-2 (145 ± 8) and IDE (292 ± 38). The level of ACE-2 and IDE stimulation was however significantly less compared with NEP, therefore indicating degree of specificity towards ECE-1 and NEP. All further studies therefore focused on the effect of K49-P1-20 on ECE-1 and NEP activity. K49-P1-20 increased the activity of ECE-1 and NEP in a concentration dependant manner. The increase in activity of both enzymes become evident at a K49-P1-20 concentration of 0.23 μM, or a peptide: enzyme molar ratio of 1:368. The high level of ECE-1 and NEP stimulation observed in response to K49-P1-20 is most likely the result of a common binding region for K49-P1-20 within these enzymes. ECE-1 and NEP in deed share 40% sequence homology^22^. However the potential sites of interaction between the enzymes and K49-P1-20 are best identified through structural biology approaches that take into account the secondary and tertiary structure of the enzymes.

Physical interaction between the activating molecule and enzyme is a common characteristic in the mechanisms of enzyme activation^23^. We used biolayer interferometry to probe possible physical interaction between K49-P1-20 and ECE-1 or NEP. N-terminal biotinylation of K49-P1-20 had no significant impact on its ability to stimulate ECE-1 activity, thus facilitating its use as a tool in research applications. Biotinylated K49-P1-20 immobilised on a streptavidin biosensor interacted directly with both ECE-1 and NEP as evidenced by the increase in response units over time. This interaction however was not observed with the biotinylated version of inverted K49-P1-20.

It is logical to assume that a conformational change that occurs following interaction with K49-P1-20 mediates the increase in enzyme activity. We investigated this by examining the effect of K49-P1-20 on the intrinsic fluorescence of ECE-1 and NEP. Fluorescence spectra of each enzyme in the presence of K49-P1-20 were distinct from that of enzyme alone. In addition, the sum of individual spectra for K49-P1-20 and ECE-1 or NEP failed to overlap with the spectra obtained by incubating K49-P1-20 with enzymes. This suggests that spectral changes that occur in the presence of K49-P1-20 is the likely result of a change in conformation of the enzymes, which in turn is a possible consequence of a direct interaction with K49-P1-20.

K49-P1-20 induced an increase in the Vmax of both enzymes reflecting the enhanced rate of substrate cleavage, whilst increasing the affinity between the substrate and ECE-1 as evidenced by the reduction in Km. However in the case of NEP an increase in both Vmax and Km was observed. This result suggests that although the specific change in conformation may reduce the affinity for the substrate, sufficient substrate availability may still increase its rate of cleavage. Therefore the specific mechanism(s) that mediate an increase in the activity may be different for both ECE-1 and NEP.

Identifying the specific residues of K49-P1-20 which are important for stimulation of enzyme activity would facilitate the development of analogs which are stable and active in biological systems. An alanine scan of K49-P1-20 identified that Leu^2^ and Ile^9^ as crucial residues for stimulation of ECE-1, while Leu^2^, Phe^3^, Glu^4^, Leu^10^, Glu^12^, Thr^13^, Lys^15^, Lys^19^ and Ser^20^ are crucial for NEP stimulation. This is also reflected in the results of Fig. 1c where K49-P1-7 and K49-P8-20 lack Ile^9^ and Leu^2^ respectively thus resulting in a significantly reduced level of ECE-1 stimulation compared to K49-P1-20.

All enzyme activity assays referred to above were conducted using a bradykinin based QFS, typically used in high throughput assays^10^,^11^,^12^,^24^. Therefore K49-P1-20 induced stimulation of ECE-1 and NEP activity was also confirmed using BigET_18-34_ and enkephalin respectively. BigET_18-34_ is a truncated version of the natural substrate of ECE-1 has previously been used in enzyme cleavage assays^12^. Aβ is a common substrate for both enzymes and its accumulation is a hall mark of Alzheimer’s disease. Previous studies have identified three N-terminal cleavage products (Aβ_1-16_, Aβ_1-17_ and Aβ_1-19_) following the digestion of Aβ by ECE-1^14^,^25^. Using LCMS, each of these products were also identified in this study, and their relative amounts were significantly higher in the presence of K49-P1-20.

Enhanced cleavage of Aβ40 not only further confirms K49-P1-20 mediated increase in ECE-1 and NEP activity, but also indicates the potential of K49-P1-20 as a pharmaceutical lead compound for the clinical management Aβ deposition. In particular, the potential implications of the discovery of K49-P1-20 for Alzheimer’s disease are highlighted by the ability of K49-P1-20 to stimulate ECE-1 activity in cerebrospinal fluid, as evidenced by the accelerated cleavage of both QFS and soluble endogenous Aβ42 (the more neurotoxic form of Aβ).

In addition to its potential as a drug lead for Alzheimer’s disease, our results show that K49-P1-20 can stimulate the enzyme activity on the surface of HEK293 cells which express NEP^25^. This in turn highlights the feasibility of using this peptide in research applications aimed at studying the cellular/biochemical effects of NEP stimulation. Addition of K49-P1-20 to HEK293 cells significantly enhanced the rate of QFS cleavage. The transient nature of the increase in rate of QFS cleavage observed is most likely the result of degradation of K49-P1-20 by other proteases expressed on the cell membrane.

In summary, here we report on the discovery of a novel peptide K49-P1-20 which significantly enhances the activity of ECE-1 and NEP as measured by the ability to cleave the bradykinin based QFS. K49-P1-20 induces a concentration dependant increase in the activity of ECE-1 and NEP while increasing the Vmax of both enzymes. Increase in enzyme activity is the likely result of a conformational change in the enzyme that occurs upon interaction with K49-P1-20. Stimulation of ECE-1 and NEP accelerated the breakdown of Aβ, thus reducing the levels of an agent associated with Alzheimer’s disease. Future studies should focus on understanding the precise mechanism of action of K49-P1-20. This would involve identifying the sites of interaction between K49-P1-20 and enzymes, precise conformational changes and how these translate to an increase in catalytic efficiency. These points are best addressed using structural biology. Understanding the mechanism of action would facilitate the synthesis of activators for other proteases which are involved in the breakdown of Aβ, thus having far reaching implications for the manipulation of Aβ levels. In addition, stimulation of enzyme activity is a poorly studied area of protease biology. As the first reported dual stimulator of ECE-1 and NEP activity, our discovery will open up a diverse array of studies in this area of enzyme biology that has to date been largely overlooked. K49-P1-20 therefore has the potential to serve both as a research tool and drug candidate in the setting of Alzheimer’s disease adding a wealth of new knowledge to the field.

## Materials and Methods

### Materials

Unless otherwise stated, all synthetic peptides used were made by Genic Bio Ltd. rhECE, NEP and IDE were purchased from R & D systems. Unless indicated appropriate dilutions were made in ECE-1 buffer (50 mM TrisCl and 150 mM NaCl, pH 6.3). ACE-2 was a generous gift from Dr. Mike Yarski (Millennium Science). Dilutions of ACE-2 were made in a buffer containing 100 mM TrisCl and 1M NaCl pH 6.5.

### Identification of K49-P1-20

#### Stimulation of ECE-1 by B. asper myotoxin II

Venoms were either commercially available (Venom Supplies Ltd, South Australia) or were a generous gift from Dr. Bryan Fry (University of Queensland). All venoms (10 ng/μL) were screened for their effects on ECE-1 (0.05 ng/μL) activity. Only the venom of *B. asper* induced a significant increase in ECE-1 activity. Initial fractionation of venom using size exclusion chromatography indicated that stimulation of ECE-1 activity was confined to the fraction containing previously identified *B. asper myotoxin II*. This toxin was further purified as described previously^8^.

*Toxin digestion*. *B. asper myotoxin II* (10 μg) was subjected to digestion by Arg C protease using standard protocols. The products of digestion were applied to a Phenomenex Jupiter C18 analytical column, and eluted using the following gradient of solvent B (90% ACN): 0–60% over 60 min. Eluted fractions were freeze-dried, reconstituted with ECE-1 buffer, and tested for effects on ECE-1 activity.

#### N-terminal sequencing

Where indicated, N-terminal sequencing of RP-HPLC fractions was performed at the Monash Biomedical Proteomics facility using previously published methods^26^.

#### Myotoxicity assay

Myotoxic effects of K49-P1-20 (25 μg/mL) was examined by monitoring the effect of K49-P1-20 on both direct twitches and resting tension of the chick biventer cervicis muscle preparation as previously described^27^.

### Enzyme activity assays

#### Assays for Endothelin Converting Enzyme-1 and NEP activity

Enzyme activities were measured using a previously described bradykinin-based quenched fluorescent substrate (QFS) assay^10^,^11^. Briefly, rhECE-1 or NEP (0.05 ng/μL) was incubated with ECE-1 buffer for 1 h at 37 °C. Where indicated, venom, *B. asper* myotoxin II or relevant synthetic peptide (10 ng/μL) was added to the reaction mixture and pre-incubated (1 h at 37 °C) with rhECE-1 or rhNEP prior to the addition of substrate ((7-methoxycoumarin-4-yl)acetyl-Arg-Pro-Pro-Gly-Phe-Ser-Ala-Phe-Lys(2, 4-dinitrophenyl); 40 μM final). The specificity of ECE-1 and NEP mediated cleavage was confirmed using the inhibitors CGS35066 (500 nM) and thiorphan (10 µM).

#### Angiotensin converting enzyme-2 (ACE-2) activity assay

Activity of ACE-2 (0.05 ng/μL) was measured using a previously described QFS based assay^28^.

#### Insulin Degrading Enzyme (IDE) activity assay

IDE was purchased from R & D Systems (Cat# 2496-ZN-010) and activity was measured as per manufacturer’s instructions.

Increase in fluorescence over time (excitation and emission wavelengths of 320 and 405 nm respectively) was taken as evidence of enzyme activity. The specific activity for each enzyme was calculated using a standard curve of known 7-methoxycoumarin concentrations. Enzyme alone (rhECE-1, rhNEP, rhACE-2 and rhIDE) was used as a control in all studies examining the effect of K49-P1-20 on enzyme activity.

#### Kinetics of enzyme activation

rhECE-1 (0.025 ng/μL) or NEP (0.05 ng/μL) was incubated for 1 h at 37 °C in the presence of K49-P1-20 (10 ng/μL or 4.6 μM). The reaction was started by adding increasing concentrations of substrate (0–120 μM).

#### Alanine scanning

A library of K49-P1-20 analogs were synthesised (Mimotopes Ltd, Clayton, Victoria, Australia) where each subsequent amino acid residue was replaced by an Ala. These analogs were initially synthesised as crude pepsets and were screened for their effects on ECE-1 and NEP activity. From these pepsets 16 analogs that induced a change in the level of ECE-1 activity were identified and resynthesised at 95% purity (Genic Bio Ltd). These 16 peptides (10 ng/μL final) were then re-screened for their effects on ECE-1 and NEP activity using the assays mentioned above.

### Interaction of K49-P1-20 with enzyme(s) and resulting conformational changes

#### BLITZ Biolayer interferometry

Interaction between K49-P1-20 and ECE-1 or NEP was studied using BLITZ Bio-Layer interferometry. Biotinylated version of native or inverted K49-P1-20 (1 μg/μg) peptide reconstituted in buffer (100 mM TrisCl and 1M NaCl; pH 6.3) was immobilised on a streptavidin biosensor through N-terminal biotin over 2 min. Following 30s equilibration with buffer, association of rhECE-1, NEP or ACE-2 (12 nM; in phosphate buffered saline) with K49-P1-20 was performed over 30 min. This was followed by a 2 min dissociation phase in buffer. Response units in the absence of K49-P1-20 (i.e. enzyme alone) was used as a blank.

#### Spectroscopy

Emission spectra were recorded at room temperature on a Perkin-Elmer LS50B luminescence spectrometer in a 1-cm path-length quartz cell. Samples were excited at 280 nm, and spectra were collected from 300–450 nm. Excitation and emission slit widths were set at 5 nm, and a scan speed of 50 nm/min was used. In all experiments the enzyme and K49-P1-20 concentrations were 0.5 μM and 8 μg/μL respectively. Four scans were performed with each sample.

### Peptide cleavage assays

#### Cleavage of BigET_18-34_ by ECE-1

ECE-1 (0.2 ng/μL) was incubated with K49-P1-20 (20 ng/μL) for 1 hr at 37 °C. In control samples, the enzyme was incubated only with buffer. Reaction was started by adding BigET_18-34_ to a final concentration of 12 ng/μL. Aliquots of equal volume were taken at T = 0, and 2 h and reaction terminated by adding TFA to a final concentration of 1%.

#### Cleavage of enkephalin by NEP

NEP (0.15 ng/μL) was incubated with K49-P1-20 (10 ng/μL) for 1 hr at 37 °C. In control samples, the enzyme was incubated only with buffer. Reaction was started by adding enkephalin to a final concentration of 12 ng/μL. Aliquots of equal volume were taken at T = 0, and 2 h and reaction terminated by adding TFA to a final concentration of 1%. All samples were stored in −80 °C until analysed by LCMS.

#### Cleavage of synthetic Aβ by activated ECE-1 and NEP

ECE-1 or NEP (0.05 ng/μL) was incubated with K49-P1-20 (100 ng/μL) for 1 hr at 37 °C. In control samples, the enzyme was incubated only with buffer. Reaction was started by adding synthetic Aβ to achieve a final concentration of 43 ng/μL. Aliquots of equal volume were taken at T = 0, and 24 h. and reaction terminated by adding TFA to a final concentration of 1%. Samples were stored in −80 °C until analysed by LCMS.

#### Cell culture

HEK293 cells (passage 10–20) grown in serum free media were centrifuged and resuspended in PBS. Cells were then seeded into a 96 well plate at a density of 6000 cells/well, and incubated at 37 °C for 2 h. Assay was started by adding QFS (40 μM) and fluorescence was monitored using excitation and emission wavelengths of 320 and 405 nm respectively. K49-P1-20 (10–500 ng/μL) or vehicle (100 mM TrisCl and 1M NaCl; pH 6.3) was added 45 min after the addition of QFS. Fluorescence was monitored for a further 50 min.

### LCMS methods

Samples were analysed by LC-MS by separation over a 10 minute gradient on a pepmap100, 75 um id, 100 Ǻ pore size, reversed phase nano column with 97% buffer A (0.1% Formic acid) to 40% B (80% Acetonitrile 0.1% formic acid), then 90% B in 3 minutes, at a flow rate of 300 nl/minute. The eluant was nebulised and ionised using the Bruker nanoESI source with a capillary voltage of 4000 V. Prior to analysis, the MicroTOFq quadrupole TOF mass spectrometer (Bruker Daltonics), was calibrated using tune mix. The LC instrument used for these studies was Ultimate 3000 nano HPLC (ThermoFisher scientific)

#### Cleavage of endogenous Aβ42 by activated ECE-1

Aβ42 levels were measured using a commercially available Human Aβ42 ELISA kit (Life technologies), according to the manufacturer’s instructions. rhECE-1 (0.04 ng/μL) alone, rhECE + K49-P1-20 (300 ng/μL) or rhECE + K49-P1-20 + CGS35066 (500 nM) was incubated at 37 °C for 1 hr. Cerebrospinal fluid (diluted 1:10 in assay buffer) from patients with Alzheimer’s disease was added to each reaction mixture. The final volume of cerebrospinal fluid added was kept to 1/10^th^ the final volume of reaction mixture, which was adjusted using ECE-1 buffer. Aliquots of equal volume were taken at 0 and 4 h, snap frozen in dry ice and stored at −80 °C until analysed by ELISA. The amount of Aβ42 remaining after 4 h was expressed as a % of initial.

### Data analysis

Non-linear regression analysis in GraphPad Prism software (version 6.04; Michaelis Menton equation) was used to calculate the enzyme kinetic parameters (Vmax and Km). In all enzyme assays the amount of substrate cleaved over 80 min was determined. Where indicated, enzyme activity in the presence of K49-P1-20 was expressed as a % of the respective control. The enzyme kinetic parameters were determined based on the rate of substrate cleavage.

Data from LCMS was processed using QuantAnalysis version 1.8 (build192). Ion chromatograms were extracted with a tolerance of 0.2Da and peak detection with a S/N threshold of 5 and smoothing width of 3. Area under the peak of interest in all LCMS chromatograms was determined using the manual integration function in Agilent Chemstation software. Data shown on all graphs represents a mean and SEM.

## Additional Information

**How to cite this article**: Smith, A. I. *et al.* N-terminal domain of *Bothrops asper* Myotoxin II Enhances the Activity of Endothelin Converting Enzyme-1 and Neprilysin. *Sci. Rep.* **6**, 22413; doi: 10.1038/srep22413 (2016).

## Figures and Tables

**Table 1:** Effect of K49-P1-20 on the Vmax and Km of ECE-1 and NEP.

	Vmax (μmols of substrate cleaved/min)	Km (μmols)
ECE-1	0.006 ± 0.001	23.9 ± 4.3
ECE-1 + K49-P1-20	0.012 ± 0.001*	16.4 ± 1.4*
NEP	0.06 ± 0.01	6.8 ± 1.8
NEP + K49-P1-20	0.31 ± 0.005*	22.4 ± 2.5*

*Significantly different compared to enzyme alone; *P* < 0.05, n = 3–4, unpaired *t*-test.
