# Corrigendum: N-terminal domain of *Bothrops asper* Myotoxin II Enhances the Activity of Endothelin Converting Enzyme-1 and Neprilysin

**DOI:** 10.1038/srep24333

**Published:** 2016-04-22

**Authors:** A. Ian Smith, Niwanthi W. Rajapakse, Oded Kleifeld, Bruno Lomonte, Nkumbu L. Sikanyika, Alexander J. Spicer, Wayne C. Hodgson, Paul J. Conroy, David H. Small, David M. Kaye, Helena C. Parkington, James C. Whisstock, Sanjaya Kuruppu

Scientific Reports 6: Article number: 22413; 10.1038/srep22413published online: 03022016; updated: 04222016

The Acknowledgements section in this Article is incomplete.

“We wish to thank William He and Steven Fernandez for technical support. This research was supported by funding from the J.o. & J.R. Wicking Trust & Mason Foundation; a strategic rant awarded to Dr. Sanjaya Kuruppu by the Faculty of Medicine Nursing & Health Sciences, Monash University; as well as funding from the Monash University’s Interdisciplinary Research Funding Scheme.”

should read:

“We wish to thank William He and Steven Fernandez for technical support. This research was supported by funding from the J.o. & J.R. Wicking Trust & Mason Foundation; a strategic grant awarded to Dr. Sanjaya Kuruppu by the Faculty of Medicine Nursing & Health Sciences, Monash University; as well as funding from the Monash University’s Interdisciplinary Research Funding Scheme. Cerebrospinal fluid samples were received from the Victorian Brain Bank Network, supported by The Florey Institute of Neuroscience and Mental Health, The Alfred and the Victorian Forensic Institute of Medicine and funded by Australia’s National Health & Medical Research Council and Parkinson’s Victoria.”

